# The importance of historical data in simulating malaria epidemiology and control interventions: application to multiple sites in Madagascar

**DOI:** 10.1186/1475-2875-13-S1-P107

**Published:** 2014-09-22

**Authors:** Emilie Pothin, Olivier Briët, Thomas Kesteman, Milijaona Randrianarivelojosia, Christophe Rogier, Thomas Smith

**Affiliations:** 1 Swiss Tropical and Public Health Institute, Basel, Switzerland; 2 Institut Pasteur de Madagascar, Antananarivo, Madagascar

## 

Planning malaria interventions requires the prediction of likely impacts of different intervention strategies. Simulation models can provide such predictions but weak information about pre-control levels of transmission, intervention coverage and access to care often makes it challenging to correctly parameterize them. We consider a number of low malaria transmission sites in Madagascar, with available historical prevalence and entomological inoculation rate (EIR) estimates (by mosquito sampling), giving disparate estimates of historical exposure. Information about implementation of Long Lasting Insecticide Nets (LLINs) and Indoor Residual Spraying (IRS), and access to healthcare, collated from malaria surveys and on-going cross-sectional studies were used to parameterise simulations of malaria transmission, prevalence and burden within the OpenMalaria platform. Multiple parameterisations were considered using various sources of data for pre-intervention transmission level, intervention coverage and access to healthcare. In some sites the simulated impact of existing vector control programs matches reasonably well the malaria prevalence measured in a recent national survey. In others it predicts lower than observed prevalence, very likely because the models do not capture residual local transmission foci. The simulations suggest that the most cost-effective vector control strategy would be to scale-up LLINs or IRS only, depending on the transmission level. Indeed, preliminary results show no additional benefit of IRS where LLINs were used. These preliminary results suggest that historical prevalence data, combined with current coverage information are potentially adequate for planning intervention strategies. The outcome of intervention scale-up is essentially unpredictable if baseline information is poor. Reproducing the observed epidemiology of malaria through simulations both provides confidence in the use of the model but serves as a basis for prospective studies that support decision-making, including cost-effectiveness analyses.

